# LuxR402 of *Novosphingobium* sp. HR1a regulates the correct configuration of cell envelopes

**DOI:** 10.3389/fmicb.2023.1205860

**Published:** 2023-07-27

**Authors:** Ana Segura, Lázaro Molina

**Affiliations:** Environmental Protection Department, Estación Experimental del Zaidín, Consejo Superior de Investigaciones Científicas, Madrid, Spain

**Keywords:** LuxR, flocculation, membrane envelop, nutrient starvation, stress

## Abstract

Although there is some evidence to suggest that LuxR-solo proteins participate in inter-species or even inter-kingdom communication, most of the LuxR-solo protein functions are unknown. We have characterized the LuxR402 regulator of *Novosphingobium* sp. HR1a, a bacterial strain with the ability to establish high numbers in the plant rhizosphere and able to degrade a wide range of polycyclic aromatic hydrocarbons. LuxR402 controls the aggregation state of the bacterial culture; cultures of a mutant strain lacking this regulator flocculate in less than 3 h without agitation. We have demonstrated that the bacterial surface of the mutant is highly hydrophobic and that the mutant cells assimilate sugars slower than the wild-type. The flocculation mechanism has been demonstrated to be involved in the survival of the strain under unfavorable conditions; the *luxR402* gene is repressed and produces flocculation in the presence of salicylate, a substrate that, although being assimilated by *Novosphingobium*, is toxic to cells at high concentrations. The flocculation of cultures in industrial setups has mainly been achieved through the addition of chemicals; these studies open up the possibility of controlling the flocculation by regulating the level of expression of the *luxR402* gene.

## 1. Introduction

Regulatory proteins of the LuxR family have been extensively studied in bacterial quorum sensing (Fuqua et al., 1994; Fuqua, 2006; Papenfort and Bassler, 2016). *luxI* and *luxR*, which encode the synthetase of the acyl-homoserine lactone, and the regulator, respectively, are frequently sequentially encoded in the bacterial chromosome (Smith and Ahmer, 2003). However, approximately 75% of the *luxR* genes annotated in the data bases in proteobacteria correspond to *luxR* solos (Hudaiberdiev et al., 2015). There is some evidence that points toward the participation of LuxR-solo proteins in inter-species and even inter-kingdom communication, but most of the LuxR-solo protein functions are unknown (Fuqua and Greenberg, 2002; Subramoni and Venturi, 2009; Brachmann et al., 2013; Brameyer et al., 2014; Bez et al., 2021).

*Novosphingobium* sp. HR1a is able to degrade low-, medium- and high-molecular weight polycyclic aromatic hydrocarbons (Segura et al., 2017), and it has shown excellent potential for rhizoremediation (Molina et al., 2021). Furthermore, this strain is able to grow in marine media (Segura et al., 2017). The ability to confront changing environments has been correlated with the number of transcription regulators in the genome (Cases et al., 2003), as these environmental bacteria have to co-ordinate the responses toward different inputs, such as temperature, oxygen tension, the presence of other organisms, antimicrobial compounds and others. In *Novosphingobium* sp. HR1a genome, 8 genes have been annotated as putative LuxR regulators in RAST server (Aziz et al., 2008), but the involvement of most of them in bacterial physiology remains unknown. Genes HWN72_19575 and HWN72_19576 are closely related to the genes encoding the canonical LuxR and LuxI proteins respectively, whilst LuxR874 (HWN72_04325) has recently been involved in plant-bacteria interactions, affecting clover root development and root colonization (Molina et al., submitted). During a preliminary screening of the phenotypes associated with the other LuxR-solos in *Novosphingobium* sp. HR1a, we observed that the knockout mutant in the *luxR*-like gene HWN72_01945 (named here *luxR402*) flocculated faster than the wild-type when cultures are in repose, without agitation. Because of the relevance of flocculation in biotechnological processes, we carried out a detailed analysis of this mutant. Our starting hypothesis is that cells of the *luxR402* knockout mutant should have their surfaces altered allowing cellular aggrupation. As LuxR402 is a regulator of the *luxR*-solo family, we thought that phenotypes, normally associated with quorum sensing (QS) regulation (i.e., motility on different surfaces), should be different between the mutant and the wild-type strains. *Novosphingobium* sp. HR1a has been described as a good colonizing strain of clover roots, and therefore, we studied if vegetal compounds, previously identified in root exudates, have an effect on growth or in the expression of the *luxR402* gene. Therefore, through hydrophobicity test, microscopy analysis, and proteomic as well as motility tests, we showed that the mutant strain has an altered cellular surface. We used transcriptomic analysis to determine the physiological status of the *luxR402* knockout mutant, identifying that the carbohydrate assimilation pathway and TCA cycle were affected in the mutant. Accordingly, the mutant presented poorer growth than the wild-type when growing with different carbon sources. Our results indicated that LuxR402 regulates the ability of *Novosphingobium* sp. HR1a to aggregate under stressful conditions as a defense mechanism against some environmental cues.

## 2. Results

### 2.1. The flocculation phenotype of the *luxR402* knockout mutant is correlated with an altered cellular morphology and surface

**Figure 1:**
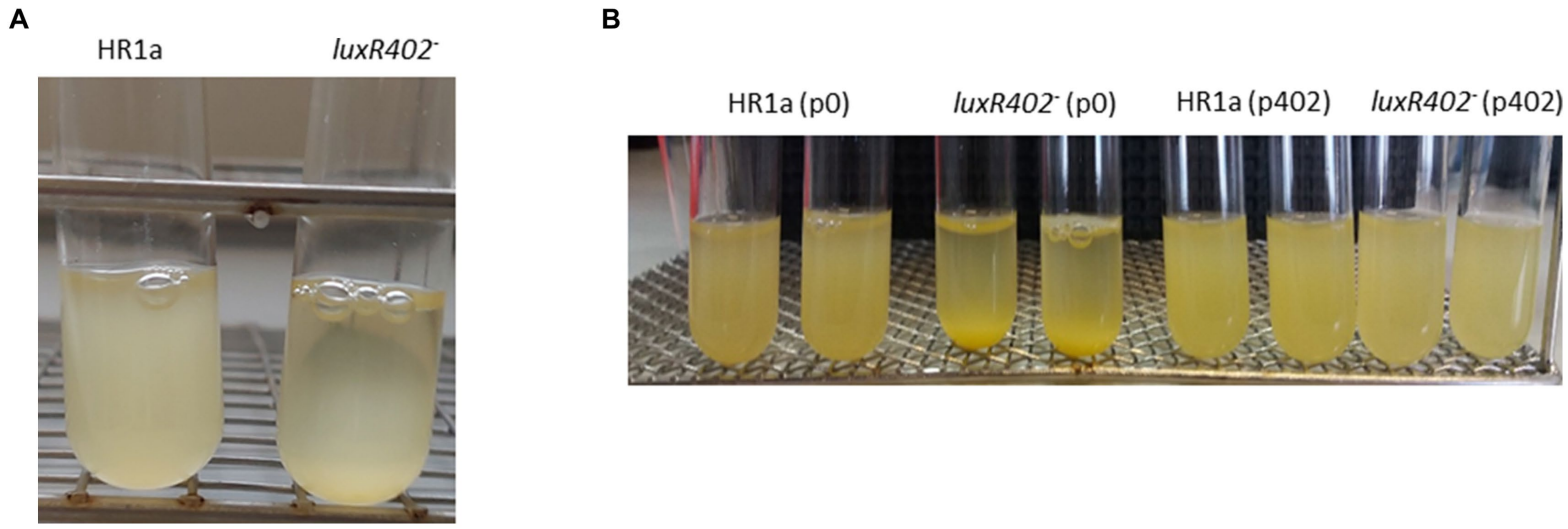
**(A)** Flocculation of *Novosphingobium* sp. HR1a *luxR402* knockout mutant in cultures grown overnight in M9 minimal medium plus glucose. The photograph was taken after 3 h without shaking. **(B)** Complementation of the *luxR402* knockout mutant phenotype. Cultures of the wild-type and mutant strains with the empty plasmid pBBR1MCS-5, HR1a (p0) and 402 (p0) respectively, and cultures of the wild-type and mutant strains with the plasmid containing *HWN72_01945*, HR1a (p402) and 402 (p402), respectively, were grown in LB media up to the stationary phase of growth. The photographs were taken 3 h after the agitation was stopped.

In liquid cultures the *Novosphingobium* sp. HR1a knockout mutant in HWN72_01945 (named *luxR402*) formed aggregates and these aggregates flocculated both in LB rich media and in M9 minimal medium with glucose as the only carbon source when the cultures were maintained without agitation. This phenotype was observable since the exponential phase of growth but became clearer after reaching the late exponential and stationary growth phase (Figure 1A). The phenotype is evident after 3 h of standstill. To demonstrate that the phenotype was specifically determined by LuxR402, we cloned the HWN72_01945 gene into the pBBRMCS5 vector (Kovach et al., 1995) under the *lacZ* promoter and we introduced the resultant plasmid into the wild-type and *luxR402* mutant strains. The complemented mutant did not flocculate as fast as the mutant (Figure 1B), although it still flocculated faster than the wild-type (not shown). Therefore, we were able to specifically assign the flocculation phenotype to the absence of a functional LuxR402.

**Figure 2:**
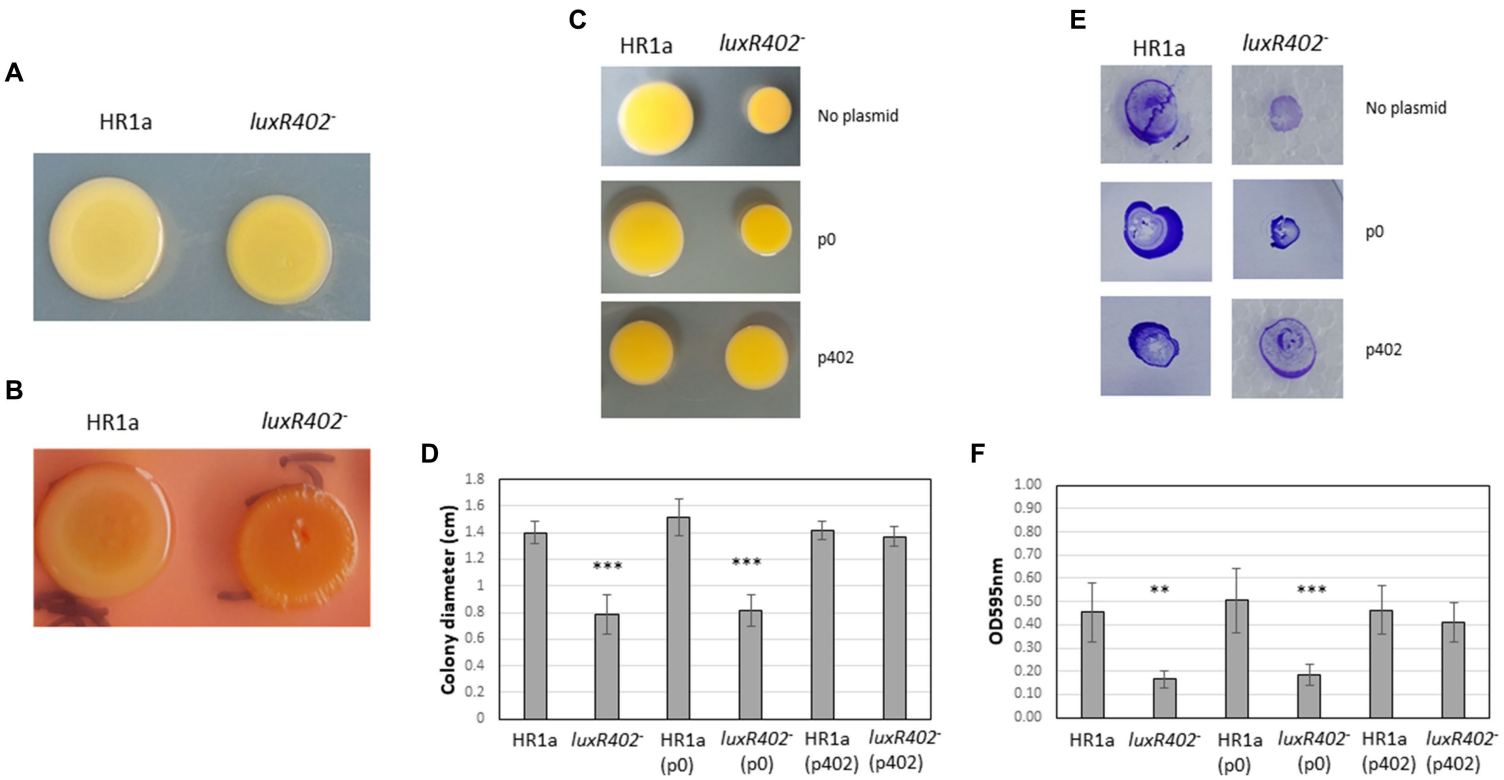
**(A)** Aspect of colonies resulting of the drop sampling of *Novosphingobium* sp. HR1a (HR1a) and of the *luxR402* mutant (402) cultivated in minimal medium M9 containing glucose as the only carbon source after 4 days at 30°C. **(B)** Aspect of the colonies in presence of Congo red under similar conditions. **(C)** Aspect of the colonies after growing for 5 days in 0.5% agar plates (swarming). **(D)** Size of the colonies (diameter) of the wild-type HR1a and mutant (luxR402) strains carrying the empty plasmid p0, or the plasmid with the *luxR402* gene (p402), after growing in 0.5% agar plates for 5 days in 0.5% agar plates. **(E)** Aspect of the colonies after growing of 5 days in 1% agar plates (twitching) upper part and the biofilm that was formed after twitching, stained with crystal violet. **(F)** Optical density measured at 595 nm of the biofilms, formed after twitching, of the wild-type (HR1a) and mutant (*luxR402^–^*) strains and the strains carrying the empty plasmid (p0) and the plasmid carrying the *luxR402* gene (p402). Asterisks indicate statistically differences between the wild-type and the other strains (***p ≤ 0.001, **p ≤ 0.01, *n* = 4–7).

Colonies of *Novosphingobium* sp. HR1a grown in plates with M9 minimal medium plus glucose had a mucoid aspect that was less visible in the *luxR402* knockout mutant (Figure 2A). Furthermore, the cellular mass in *Novosphingobium* sp. HR1a colonies was slippery when manipulated, whilst in the mutant strain it was stickier. These results suggest that LuxR402 was involved in the polysaccharide production. In an attempt to characterize the nature of the polysaccharide, we added Congo red to the plates, a dye that binds to some β-D-glucans, dyeing the colonies with an intense red color. As shown in Figure 2B, the wild-type colony did not take on a strong red color and the mutant strain presented only a slightly more intense red color, suggesting that β-glucans were not the main exopolysaccharides in any of the strains. Neither *Novosphingobium* sp. HR1a nor the *luxR402* knockout mutant swam in 0.3% agar plates (not shown). Typical swarming was not observed in the wild-type strain when grown in 0.5% agar plates, although the mutant colony presented a smaller diameter than the wild-type (Figures 2C,D). Twitching and biofilm formation after twitching was more evident in the wild-type than in the mutant strain (Figures 2E,F).

**Figure 3:**
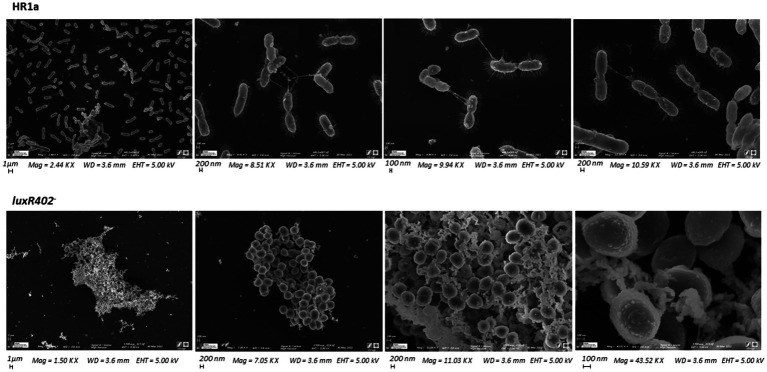
Scanning microscopy photographs of the wild-type and *luxR402* knockout mutant strains after being cultivated in liquid M9 minimal medium plus glucose up to the exponential phase of growth.

When the wild-type and *luxR402* mutant strains were analyzed under scanning microscopy, we observed that while the cells of *Novosphingobium* sp. HR1a presented the typical bacillary morphology, the mutant cells presented a coccoid morphology (Figure 3). The cells of mutant *luxR402^−^* were visualized as aggregates whilst wild-type cells were observed as independent units that, in many cases, were under division, suggesting that they were actively growing. Furthermore, in the mutant strain, cell size was smaller, extracellular material could be observed adhered to the cell envelop, whilst in the wild-type strain appendages (probably pili and/or fimbriae) could be seen.

**Figure 4:**
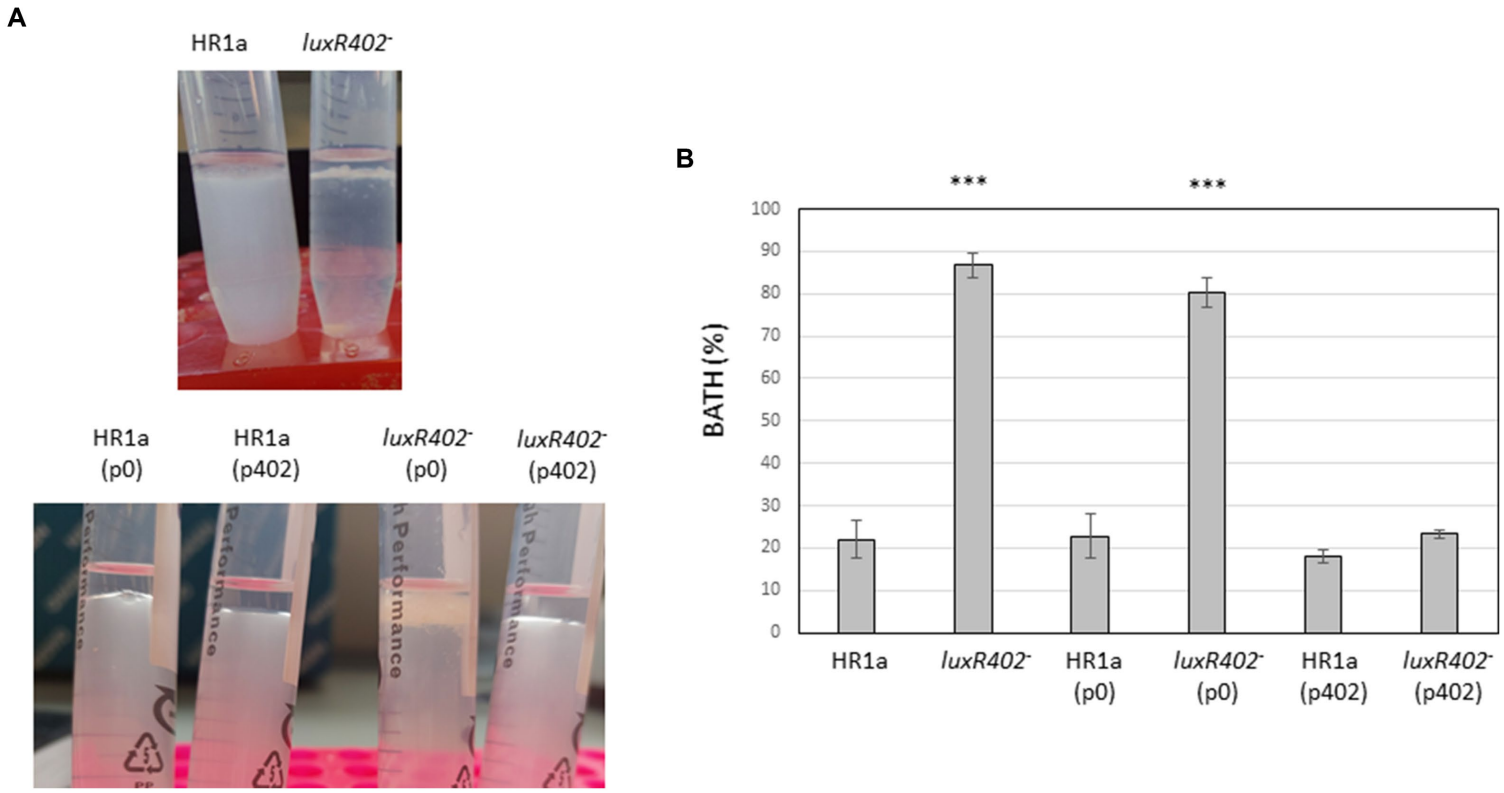
**(A)** Photograph of the cultures after being exposed to *n*-hexane 10 min after agitation. **(B)** BATH values in the wild-type and mutant strains and in the strains carrying the empty plasmid p0 and the plasmid encoding the *luxR402* gene (p402). ***indicate statistically differences between the wild-type and the other strains (*p* ≤ 0.001; *n* = 4).

To study the hydrophobicity of the cellular surfaces, we analyzed the bacterial adhesion to the hydrocarbons (BATH). After exposition to *n*-hexane, most of the mutant cells remained in the interphase between the water and organic solvent layers, whereas most of the wild-strain cells remained in the water layer (Figure 4), indicating that cells from *Novosphingobium* sp. HR1a were more hydrophilic (BATH = 22 ± 9.5%) than those of the *luxR402* knockout mutant (BATH = 86.7 ± 2.7%).

All these phenotypes indicate that LuxR402 has an important role in the reduction of the flocculation, in the cellular and colony morphology and in external surface hydrophobicity in *Novosphingobium* sp. HR1a.

### 2.2. The lack of LuxR402 modifies the protein content of the cell surface of *Novosphingobium* sp. HR1a

**Figure 5:**
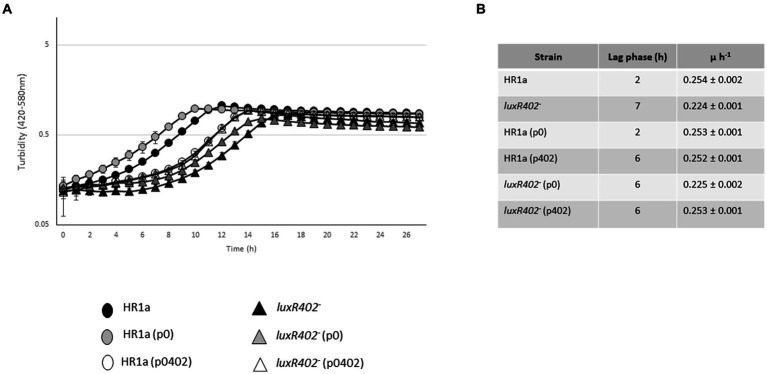
**(A)** Growth of *Novosphingobium* sp. HR1a strains (circles) and mutant *luxR402^–^* strains (triangles) in M9 minimal medium plus glucose. Black symbols correspond to strains without plasmids; grey symbols to strains with pBRRMCS5, and white symbols to strains expressing the *luxR402* gene. **(B)** Table with the lag phases and growth rate of the different strains. Growth was measured with the Bioscreen device.

**Figure 6:**
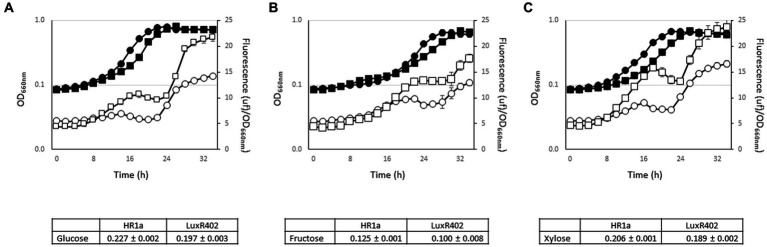
**(A)** Growth (black symbols) and expression from the *luxR402* promoter, measured as relative fluorescence of GFP (empty symbols) of *Novosphingobium* sp. HR1a (*Prlux402*-GFP) (circles) and *luxR402* (*Prlux402*-GFP) mutant (squares) in **(A)** glucose, **(B)** fructose and **(C)** xylose as the sole carbon source. Tables showed the growth rate (μ h^−1^) of the wild-type and mutant strains in each carbon source. Error bars indicate the standard deviation of the six experiments carried out in microplates. Growth and fluorescence were measured with a Varioskan LUX Multimode Microplate Reader.

Given the cell surface differences that we observed between the wild-type strain and the *luxR402* knockout mutant, we carried out a proteomic analysis to identify secreted proteins or proteins attached to the outer membrane, by analyzing the proteins released to the media after vigorous agitation. Peptides that mapped within ribosomal and other cytoplasmic proteins were identified at low levels in the wild type (3 ± 0.27%) and mutant *luxR402* (5 ± 3.6%) cultures, suggesting that partial cellular lysis during the procedure or during the growth culture had occurred. However, there were no significant differences between the two strains, suggesting that cell integrity was not more compromised in the mutant than in the wild-type strain. Thirty-nine proteins were significantly differentially identified. Two proteins were less expressed in the wild-type strain than in the mutant; these proteins were involved in carbohydrate metabolism (Table 1). An over-representation of proteins related with oxidative phosphorylation (carbohydrate uptake, ATP synthase, cytochrome related proteins, succinate dehydrogenase, amongst others) was observed in the wild-type in comparison with the mutant strain. The decreased expression of these proteins in the mutant strain correlated with the longer lag phase observed when glucose was used as the sole carbon source. To unequivocally assign a role to the LuxR402 regulator in this phenotype, we complemented the mutant strain with p402. When we introduced the plasmid expressing the *luxR402* gene in the mutant strain, the lag phase was similar in the mutant and complemented strains, however, growth rate was higher in the complemented strain than in the mutant strain (Figure 5B) and similar to that of the wild-type strain (Figure 5). The introduction of plasmid expressing *luxR402* in the wild-type strain also affected the growth, increasing the lag phase of the culture. Therefore, the modifications in the level of expression of the *luxR402* gene during exponential phase of the growth clearly affect the growth of *Novosphingobium* sp. HR1a in M9 minimal media. We tested the growth of the bacteria in 17 different carbon sources and in all the cases, the mutant clearly presented a longer lag phase than the wild-type strain, and, a slightly lower growth rate (Figures 5, 6 and Supplementary Figure S1).

**Table 1:** List of proteins identified with different number of peptides when comparing wild-type *vs luxR402.*

ID (*N. resinovorum*)	Locus	Avge #pept HR1a	Avge #pept 1709	Log_2_FC	Name	Function	Signal peptide	Location
BES08_00045	HWN72_03495	0.3	6.9	−4.38	Poly(beta-D-mannuronate) lyase	Carbohydrate metabolism/polymer degradation/alginate degradation	0	Cell envelope/periplasmic
BES08_21170	HWN72_20700	2.0	10.0	−2.44	Glycoside hydrolase family 3/Beta glucosidase	Carbohydrate metabolism	(Sec/SPI)	Cell envelope
BES08_21280	HWN72_20595	35	6.5	2.43	Carbohydrate-selective porin OprB	Transport/carbohydrate RpfN protein/sugar transport	(Sec/SPI)	Cell envelop/outer membrane
BES08_12905	HWN72_15740	26.7	4.3	2.62	OmpW family protein	Transport/small hydrophobic substances	(Sec/SPI)	Cell envelop/outer membrane
BES08_10130	HWN72_13005	23	1.3	4.1	Multimodular peptidoglycan glycosyltransferase (EC 2.4.1.129) (EC 3.4.-.-)	Peptidoglycan biosynthesis	0	Cell envelope
lptD	HWN72_09055	12	0.4	4.8	LPS-assembly protein LptD	Transport/ involved in the assembly of lipopolysaccharide (LPS) at the surface of the outer membrane.	(Sec/SPI)	Cell envelop/outer membrane
murG	HWN72_12615	7	0	–	UDP-N-acetylglucosamine--N-acetylmuramyl-(pentapeptide) pyrophosphoryl-undecaprenol N-acetylglucosamine transferase (EC 2.4.1.227)	Peptidoglycan biosynthesis	0	Cell membrane and cytosol
BES08_03260	HWN72_06745	16.0	2.0	2.88	Peptidyl-prolyl cis-trans isomerase PpiD (EC 5.2.1.8)/Parvulin-like PPIase	Transport/proteins/ Chaperone that functions as a gatekeeper on the periplasmic side of the SecYEG translocon	0	Cell envelope/periplasmic site
BES08_17015	HWN72_02460	19.7	4.8	2.04	TonB-dependent receptor	Membrane energization	(Sec/SPI)	Cell envelop/outer membrane
BES08_21360	HWN72_20510	9.6	1.7	2.48	TonB-dependent receptor-like protein/ferrichrome-iron receptor	Membrane energization	(Sec/SPI)	Cell envelop/outer membrane
BES08_19360	HWN72_24145	7.7	0.4	4.1	TonB-dependent receptor, mostly Fe transport/related with ribokinase	Membrane energization	(Sec/SPI)	Cell envelop/outer membrane
BES08_09450	HWN72_12310	14.3	3.0	2.24	MipA/OmpV family protein	Transport/protein chaperone involved in the biogenesis of (OMPs)/Structural protein MipA	0	Cell envelop/outer membrane
0BES08_03610	HWN72_07090	11. 7	0	–	Hypothetical protein/related with outer membrane protein assembly factor YaeT precursor	Transport/protein secretion/	0	Secreted?
secD	HWN72_09085	10	0	–	Protein-export membrane protein SecD (TC 3.A.5.1.1)	Transport/proteins	0	Cell envelop/inner membrane
BES08_15415	HWN72_00770	11.7	1.3	3.2	Flagellar motor protein MotA/	Transport/proteins	0	Cell envelop/inner membrane
BES08_12135	HWN72_15095	8	0.4	4.2	Flagellar motor switch protein FliG	Cell motility	0	Cell envelop/inner membrane
BES08_28190	HWN72_28990	7	0	–	Flagellar hook-length control protein FliK/autotransporter	Cell motility	(Sec/SPI)	Cell envelop/outer membrane
BES08_01550	HWN72_05020	10.3	2.2	2.25	LemA family protein	Signalling/sensoring the external medium	(Sec/SPII)	Cell envelope
rpoB	HWN72_00120	24.0	3.5	2.79	DNA-directed RNA polymerase subunit beta	Transcription	0	Cytosol
rpoC	HWN72_00115	22.0	3.0	2.86	DNA-directed RNA polymerase subunit beta’	Transcription	0	Cytosol
BES08_04640	HWN72_08145	8.3	0	–	DNA recombination protein RmuC	DNA recombination	0	Cell envelope
rpoA	HWN72_12000	8. 7	0.4	4.3	DNA-directed RNA polymerase alpha subunit (EC 2.7.7.6)	Transcription	0	Cytosol
infB	HWN72_03135	10	0.4	4.5	Translation initiation factor 2	Protein synthesis	0	Pseudogen
atpD	HWN72_08805	19.3	2.6	2.89	ATP synthase subunit beta	Respiratory chain/phosphorilation/ATP synthesis	0	Cell envelop/inner membrane
atpA	HWN72_08795	16. 7	0.9	4.3	ATP synthase alpha chain (EC 3.6.3.14)	Respiratory chain/phosphorilation/ATP synthesis	0	Cell envelop/inner membrane
atpG	HWN72_08800	9. 7	0	–	ATP synthase gamma chain (EC 3.6.3.14)	Respiratory chain/phosphorilation/ATP synthesis	0	Cell envelop/inner membrane
BES08_09995	HWN72_12870	17.0	3.0	2.49	Phosphoenolpyruvate carboxylase (EC 4.1.1.31); divergent?	Carbohydrate metabolism/TCA cycle	0	Cytosol
BES08_13365	HWN72_16210	10. 7	0	–	RND multidrug efflux transporter; Acriflavin resistance protein	Transport/efflux	0	Cell envelop/inner membrane
BES08_00190	HWN72_03645	9	0	–	Outer membrane autotransporter barrel	Transport/proteins	(Sec/SPI)	Secreted?
BES08_10115	HWN72_12990	11	0	–	Peptidase/ HflK protein	Cell division/Protein metabolism	0	Cell envelop/periplasmic
ftsH	HWN72_12990	6. 7	0	–	ATP-dependent zinc metalloprotease FtsH Cell division protein FtsH (EC 3.4.24.-)	Cell division	0	Cell envelop/periplasmic
BES08_11415	HWN72_14195	12	0	–	DNA translocase/Cell division protein FtsK	Cell division	0	Cell envelop/inner membrane
ftsZ	HWN72_12580	13. 7	0	–	Cell division protein FtsZ (EC 3.4.24.-)	Cell division	0	Cell envelop/inner membrane
BES08_16530	HWN72_01905	7	0	–	Hypothetical protein/related heme biosynthesis	Oxidative stress?	0	Cell envelope
BES08_27855	HWN72_28660	7. 7	0	–	Catalase (EC 1.11.1.6)	Oxidative stress	0	Cytosol
edd	HWN72_12855	14	0	–	Phosphogluconate dehydratase (EC 4.2.1.12)	Carbohydrate metabolism; Entner-Doudoroff pathway.	0	Cytosol
sdhA	HWN72_10715	15.3	0.9	4.1	Succinate dehydrogenase flavoprotein subunit (EC 1.3.99.1)	Carbohydrate metabolism/TCA cycle	0	Cell envelop/inner membrane
BES08_13530	HWN72_16380	7. 3	0	–	L-sorbosone dehydrogenase	Carbohydrate metabolism/oxidative stress		
BES08_15860	HWN72_01220	10. 7	0.4	4.6	Mlr7403 protein/ TPR_21 domain-containing protein	Unknown	0	Cell envelope

The number of peptides was normalized with the dried weight of the cultures.

A number of peptidoglycan biosynthesis proteins and a protein involved in the transport/assembly of lipopolysaccharide proteins were identified at lower levels in the mutant than in the wild-type suggesting that the cell envelop differs between wild-type and mutant strains, supporting the hypothesis about the involvement of *luxR402* gene in the control of the cell surface composition.

### 2.3. *luxR402* is expressed at the stationary phase of growth in the wild-type strain, but it is transitorily expressed during exponential phase in the mutant strain

To study the expression from the *luxR402* promoter throughout the growth phase, we constructed a biosensor in which the *luxR402* promoter drove the expression of the green fluorescent protein (GFP). The expression from the *luxR402* promoter increased significantly at the stationary phase of growth when grown with glucose as the only carbon source (Figure 6A). In the absence of a functional LuxR402 protein, the expression from the *luxR402* promoter started to increase at the beginning of the exponential phase of growth, to remain constant until the beginning of the stationary phase of growth where the *luxR402* promoter became active again (Figure 6A). The expression level in the mutant strain at the stationary phase of growth was higher than in the wild-type strain. These results suggest that LuxR402 regulates their own expression (Figure 6A). This expression is coherent with the flocculation phenotype of the mutant strain that started to be observable from the beginning of the exponential phase of growth. Similar induction patterns were observed when the strains were grown with fructose (Figure 6B) or xylose (Figure 6C) as the sole carbon source, although in xylose the expression levels of the *luxR402* promoter were higher than in the other sugars.

### 2.4. The carbohydrate metabolism, the TCA cycle and the oxidative phosphorylation of the *luxR402* knockout mutant was altered during the exponential phase of growth

To reveal the effect that the regulatory protein *luxR402* had over the gene expression when growing in glucose and because of the different expression of the *luxR402* promoter during the exponential phase of growth of the wild-type and mutant strains, we carried out a transcriptomic analysis during this phase of growth. We found that 411 genes were up-regulated (*p*_adj_ < 0.05) in the mutant strain, whilst 644 were down-regulated (Supplementary Figure S2A and Supplementary Table S1). The gene ontology (GO) and KEGG pathway analysis (Supplementary Figure S2B) suggest that carbohydrate metabolism, signal transduction and transport functions are mainly altered in the mutant strain. Amongst the down-regulated genes, those related with carbon metabolism, TCA cycle, biosynthesis of secondary metabolites and oxidative phosphorylation were the most abundant (Supplementary Figure S2C), whilst the genes related with two component regulatory systems, peptidoglycan synthesis and ribosome were up-regulated in the mutant (Supplementary Figure S2D).

Only five genes were down-regulated with log_2_FC ≤ −2 in the mutant when compared with the wild-type strain (Table 2). Four of these genes (*HWN72_20595* to *HWN72_20610*) encoded functions related with carbohydrate transport and utilization and with the phosphotransferase transport system (PTS). HWN72_20595 (putative OprB) was also previously identified in our proteomic analysis (Table 1), supporting the transcriptomic results. The decreased expression of the sugar transport and PTS system in the mutant strain, correlated with the longer lag phase previously observed previously in different carbon sources (Figures 5, 6 and Supplementary Figure S1).

**Table 2:** List of genes up- or down-regulated in the mutant strain when compared with the wild-type.

Gene_id	Log_2_fold change	Gene_description
HWN72_20595	−5.14	Carbohydrate porin OprB family
HWN72_20600	−4.94	PTS fructose transporter subunit EIIB/C
HWN72_20605	−4.28	1-phosphofructokinase (*pfkB*)
HWN72_20610	−2.94	Phosphoenolpyruvate–protein phosphotransferase 2 (*ptsP*)
HWN72_14810	−4.69	Hypothetical protein
HWN72_14815	−1.82	Polysaccharide biosynthesis tyrosine autokinase
HWN72_14090	3.45	homocysteine S-methyltransferase family protein
HWN72_14095	3.25	GNAT family N-acetyltransferase
Novel00673	2.10	Vitamin B12 dependent methionine synthase, activation domain
HWN72_01945	2.99	Bacterial regulatory proteins, luxR family (l*uxR402*)
HWN72_23475	2.38	TonB-dependent receptor
HWN72_17540	2.37	LLM class flavin-dependent oxidoreductase
HWN72_21935	2.17	DUF692 domain-containing protein
HWN72_18160	2.10	Nuclear transport factor 2 family protein
HWN72_21235	2.04	tRNA-Ser
HWN72_18065	2.01	Efflux RND transporter periplasmic adaptor subunit

Gene *HWN72_14810* encodes a hypothetical protein but contiguous to this gene, probably forming an operon, gen *HWN72_14815* encoded a putative polysaccharide biosynthesis tyrosine auto-kinase, whose expression is down-regulated with a log_2_FC of −1.82 (Table 2). Ten genes were up-regulated in the mutant strain, amongst them, genes involved in the interconversion of methionine and cysteine, and the *luxR402* gene, confirming the previous results about their own regulation (Figure 6).

### 2.5. *luxR402* promoter responded to the external addition of N-(3-hydroxyoctanoyl)-DL-homoserine lactone and aromatic compounds

**Figure 7:**
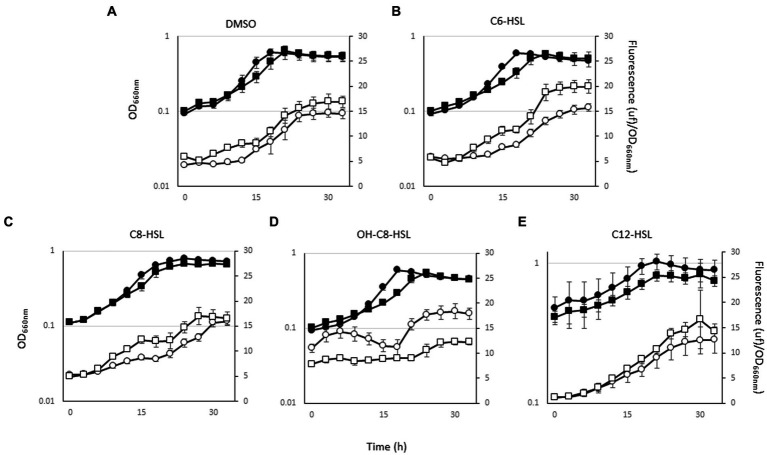
Growth (black symbols) and GFP expression from the *luxR402* promoter (open symbols) of the wild-type strain (circles) and mutant strain (squares) in the presence of: **(A)** DMSO; **(B)** *n*-hexanoyl-DL-homoserine lactone (C6-HSL); **(C)** *n*-octanoyl-DL-homoserine lactone (C8-HSL); **(D)** *n*-3-hydroxyoctanoyl-DL-homoserine lactone (OH-C8-HSL); **(E)**; *n*-dodecanoyl-DL-homoserine lactone (C12-HSL).

LuxR solo proteins have been involved in the interspecies and/or inter-kingdom communication. To demonstrate the implication of LuxR402 in this communication, we analyzed the expression of the *luxR402* promoter after the addition of different homoserine lactones (AHLs), and different signals of plant stress. The *luxR402* promoter responded to the external addition of N-(3-hydroxyoctanoyl)-DL-homoserine lactone; there was an increase in the expression of the GFP during the exponential phase of growth in the wild-type strain, whilst no such increase was observed in the *luxR402* mutant (Figure 7D). There was not response when *n*-hexanoyl-L-homoserine lactone, *n*-octanoyl-L-homoserine lactone, or *n*-dodecanoyl-L-homoserine lactone were added to the media (Figures 7A, 7B, 7C and 7E).

**Figure 8:**
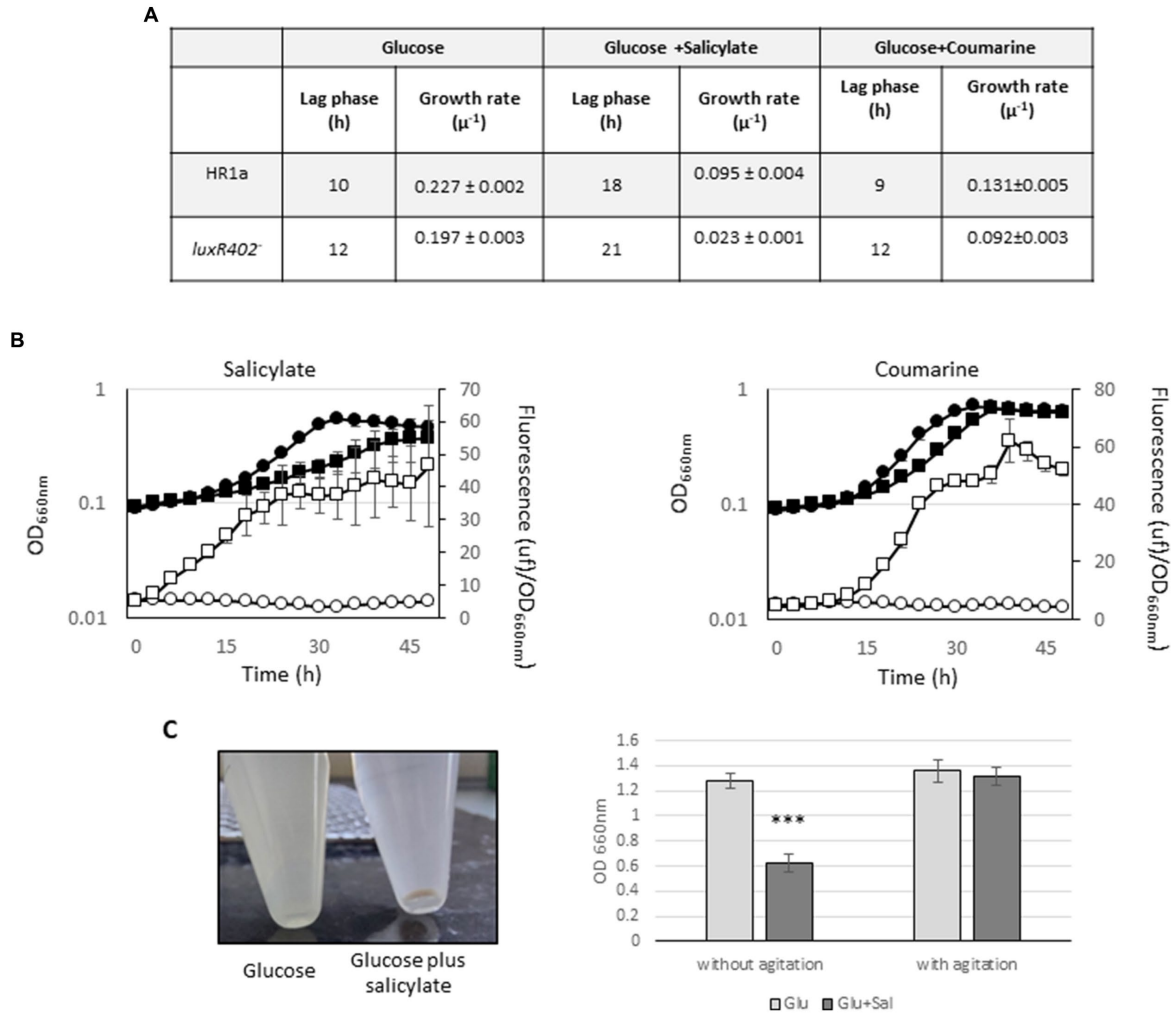
**(A)** Table with the lag phase and growth rate of the cultures of the wild-type (HR1a) and mutant (*luxR402^–^*) strains in glucose, glucose plus 5 mM salicylate and glucose plus 1 mM coumarine. **(B)** Growth (black symbols) and GFP expression from *luxR402* promoter (empty symbols) of cultures of the wild-type strain (circles) and *luxR402* knockout mutant (squares) growing with glucose plus 0.5 mM salicylate glucose plus 1 mM coumarin. **(C)** Flocculation of *Novosphingobium* sp. HR1a with salicylate: cultures at exponentially phase of growth (DO_660nm_ ≈ 0.8) were challenged with 5 mM of salicylate and incubated with agitation for 3 h; photography was taken after 16 h without agitation. OD data was taken after 16 h without agitation or after 16 h after vortexing the culture. Statistical analysis was done comparing the OD of the HR1a strain in the presence/absence of salicylate (***indicate *p* < 0.001; *n* = 4).

We also tested the effect of salicylate, a plant-derived compound involved in plant responses against certain types of stress (Raza et al., 2022), hormones (jasmonic acid, indole acetic acid) and other vegetal compounds (phenylacetate, phenylactate, coumarine, γ-amino butyric acid or protocatechuic acid) as inductors of the expression of the *luxR402* gene. No effect was observed with most of compounds tested (not shown), however we did perceive a clear effect in the presence of salicylate and coumarine. The presence of 5 mM salicylate retarded the growth of both strains when compared with growth with only glucose (Figure 8A); this delay in growth was stronger in the *luxR402* knockout mutant than in the wild-type (Figures 8A,B). Furthermore, expression from the *luxR402* promoter was totally repressed in the wild-type strain, whilst the expression in the mutant strain was induced (Figure 8B). The maximal relative fluorescence from the *luxR402* promoter in the mutant strain in the presence of salicylate was ≈ 40 units/OD_660nm_ at 36 h (Figure 8B) whilst in the absence of this compound was around 22 units/OD_660nm_ at 34 h (Figure 6A). The growth and expression pattern was similar when we added 1 mM coumarin, another plant defense metabolite, to the culture (Figure 8C). We have previously observed that cultures of strain HR1a were not able to grow in M9 minimal media with 5 mM salicylate, however they did grow if salicylate was added at 1 mM every 12 h (not shown). To further examined this process, we add 5 mM of salicylate into a glucose-grown culture after 6 h of growth and we culture the cells another 3 h. Then we let the culture over the table for 13 h. After this time, the flocculation of the *Novosphingobium* sp. HR1a culture was observed (Figure 8C).

### 2.6. The knockout *luxR402* mutant is less resistant to the external addition of H_2_O_2_

**Figure 9:**
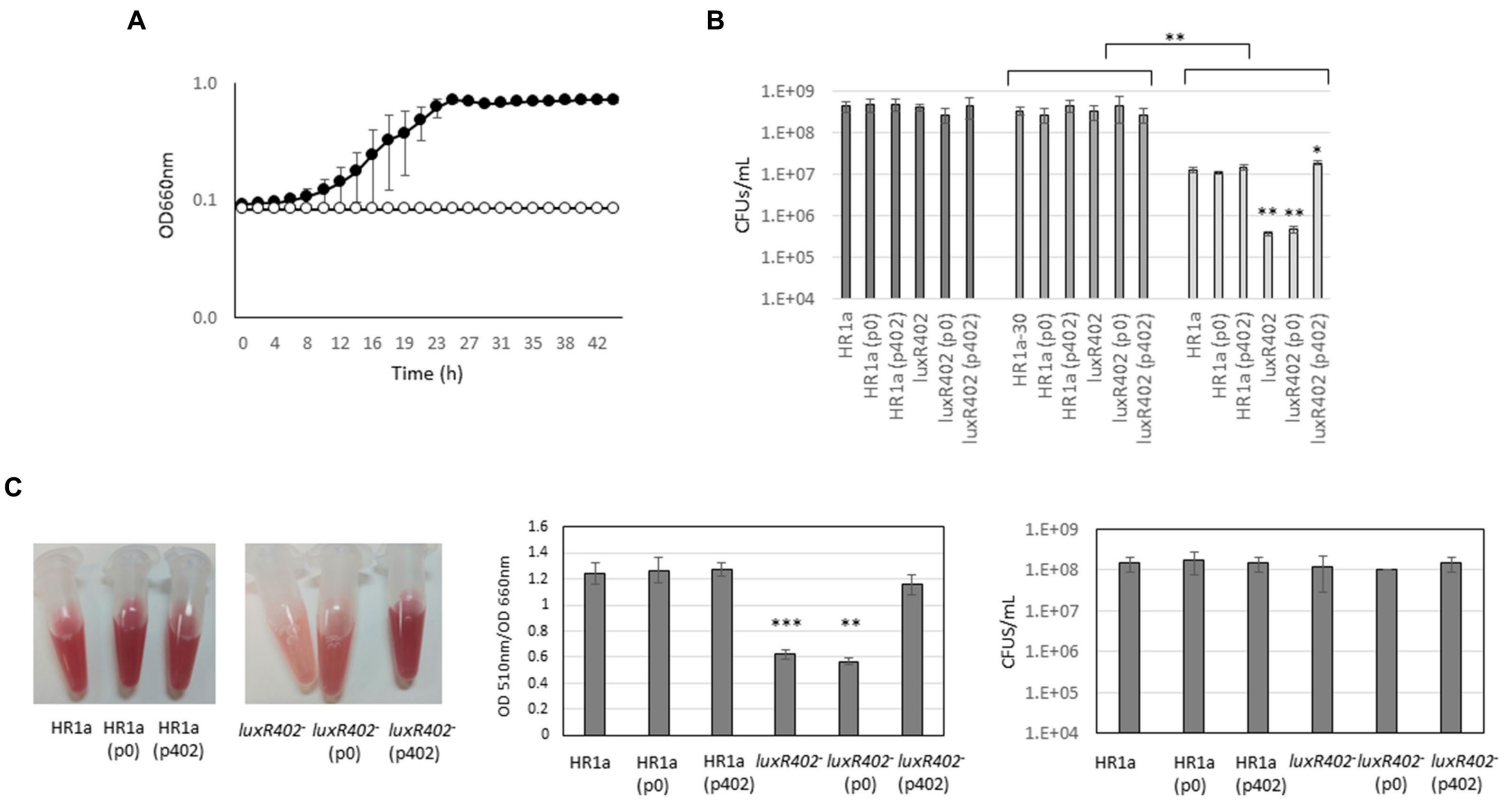
**(A)** Growth curves of *Novosphingobium* sp. HR1a (black circles) and *luxR402* knockout mutant (white circles) in M9 minimal media with glucose as the sole carbon source and in the presence of 0.5 mM H_2_O_2_. **(B)** Survival of the wild-type, *luxR402* knockout mutant, and complemented strains 30 min after addition of 0.5 mM of H_2_O_2_. Dark grey bars indicate the number of CFUs in the different cultures before H_2_O_2_ addition (*t* = 0); medium dark grey bars indicate the number of CFUs at time 30 min and light grey bars the number of CFUs 30 min after H_2_O_2_ addition. There were no statistically significant differences among the initial number of CFUs in the cultures at the two different times without addition of H_2_O_2_ (Kruskal-Wallis One Way Analysis of Variance on Ranks, *n* = 6). *t*-test analyses were done for comparison of the number of CFUs of the same strain with and without H_2_O_2_ (asterisks in the upper part of the figure) and of the different strains versus wild-type when challenged with H_2_O_2_ (asterisks within the figure). **indicate *p* ≤ 0.01 and *indicate *p* ≤ 0.05; *n* = 4. **(C)** Analysis of the specific TTC-reducing activity of the cultures using TTC and number of CFUs at the time of the analysis.

The cell envelope is crucial for resistance to stress, and as *luxR402* presented clear differences in cell surface when compared with the wild-type, we analyzed the growth of the two strains toward hypo- and hypersaline stress, the presence of antibiotics (ampicillin and chloramphenicol) and oxidative stress. Growth was not significantly affected by either hypo-, or hypersaline stress, or antibiotics, however, the growth of the *luxR402* was clearly affected in the presence of 0.5 mM of H_2_O_2_ (Figure 9A). In fact, the presence of this concentration of H_2_0_2_ also produced a decreased in the growth rate in the wild-type (from 0.227 ± 0.002 μ^−1^ in the absence of this compound to 0.127 ± 0.01 μ^−1^ in the presence of H_2_0_2_).

To further investigate this phenotype, we grew the cultures until they reach an O.D. at 660 nm of 0.5; then we added 0.5 mM of H_2_O_2_ and we measured the survival after 30 min (Figure 9B). In the wild-type strain, we observed a decrease in the CFU numbers of around 1.5 orders of magnitude. The presence of the empty plasmid (p0) or of the plasmid with the *luxR402* gene (p402) did not modify these numbers. However, in the mutant strain this decreased was of 3 orders of magnitude; the presence of the empty plasmid did not modify the number of CFUs after the shock, however, the expression of the *luxR402* from the plasmid did increase the number of survival cells up to the same numbers than the wild-type strain (Figure 9B). The capacity of the different strains to reduce 2,3,5-triphenyl-2H-tetrazolium chloride (TTC) in presence of oxygen peroxide (0.25 mM) was higher in wild type than in the mutant strain, and this phenotype was complemented by plasmid p402 (Figure 9C). However, the number of CFUs under these conditions was not affected in the mutant when compared with the wild-type strain. These results suggested that, under these conditions, the specific TTC-reducing activity of the wild-type strain was higher than that in the mutant, and, again, this phenotype was complemented by the expression of the *luxR402* gene.

## 3. Discussion

### 3.1. The *luxR402* mutant cells flocculated and had an altered envelop

LuxR-solos are regulatory proteins that are thought to be involved in cell–cell communication, although not many LuxR-solos have been functionally characterized. We have identified a LuxR-solo protein, named LuxR402, which controls the auto-aggregation capacity of *Novosphingobium* sp. HR1a. Auto-aggregation has been described as a common strategy to skip the deleterious effects of certain environmental clues, such as the presence of toxic compounds, antibiotics, lack of nutrients and oxidative stress (Trunk et al., 2018). Although auto-aggregation is a common phenomenon in bacteria, it is still poorly understood. Curli, fimbriae, exopolysaccharides, DNA and extracellular proteins have been described as structures that may facilitate aggregation (Salehizadeh and Shojaosadati, 2001; Sun et al., 2015; Nouha et al., 2018). *Novosphingobium* sp. HR1a did not auto-aggregate under normal laboratory conditions, however, in the absence of the LuxR402 protein, cells formed clumps and finally flocculated.

Four lines of evidence indicated that the cellular surface in the mutant had very different properties when compared with the wild-type strain. The first evidence came from the different aspect of the bacterial colonies and the lack of stain with Congo red. The second line of evidence was related with the differences in motility; twitching motility (flagellar-independent motility) was reduced in the wild-type strain. Furthermore, although we did not visualize the typical rafts (Römling, 2005) formed during swarming in 0.5% agar plates in the colonies of the wild-type strain, the colony morphology of the *luxR402* knockout mutant was clearly different. We cannot confirm that this behavior is related with swarming, but it clearly points to different properties in the cell surface. The third line of evidence came from the different morphologies of the cells that we observed under the microscope; whilst the wild-type cells presented the typical bacillary morphology of *Novosphingobium* (Liu et al., 2005; Li et al., 2017; de Lise et al., 2019), the mutant strain cells were coccoid. Finally, the hydrophobicity of the *luxR402* knockout cells was higher than that of the wild-type strain.

Although some LuxR-like proteins have been previously seen to be involved in flocculation in *Azospirillum brasilense*, *Azorhizobium caulinodans* ORS571 and *Sinorhizobium melitoti* (Pereg-Gerk et al., 1998; Sorroche et al., 2010; Liu et al., 2020), in these organisms, contrary to what we have observed, the lack of the protein causes the absence of flocculation. The AclR1 of *Azorhizobium caulinodans* ORS571 has been involved in the positive regulation of cell motility and flocculation, whilst this protein is negatively regulates the exopolysaccharide production and biofilm formation (Liu et al., 2020). ExpR is a LuxR-type regulator that controls several functions in *S. meliloti*, amongst them, the exopolysaccharide I (EPS I) composed of succinoglycan and EPS II (galactoglucan) (Sorroche et al., 2010).

### 3.2. LuxR402 senses sugar availability in the medium and participates in sugar uptake and degradation

Although LuxR402 has the typical length of a LuxR protein (291 amino acids) and the conserved helix-turn-helix (HTH) domain, it only has conserved three out of the seven amino acids of the AHL binding domain. However, we have determined that this protein responds to the addition of external *of N-*(3-hydroxyoctanoyl)-DL-homoserine lactone, and that the pattern of expression is typical of the LuxR-regulators, and is expressed early on in the stationary phase of growth and is auto-regulated. One of the main differences in the *luxR402* knockout mutant when compared with the wild-type strain (besides the higher level of expression of the *luxR402* promoter) is the fact that *luxR402* is transitorily expressed during the exponential phase in the mutant strain. The transcriptomic assays, carried out during this phase of growth when grown in glucose, reveal that the genes involved in sugar uptake and metabolism were down-regulated in the mutant. Our physiological studies indicate that in fact, the mutant strain grew more slowly in glucose than the wild-type strain. It has been described that under non-favorable growth conditions, bacterial cell surfaces become more hydrophobic, thus facilitating aggregation (Kjelleberg and Hermansson, 1984). The transition from bacillary forms to coccoid morphologies that formed biofilms under low-nutrient conditions has been described in *Acinetobacter* sp. (James et al., 1995). Therefore, it is conceivable that in the absence of a functional LuxR402, and through the decreased expression of the *HWN72_20595, HWN72_20600* and *HWN72_20605*, genes involved in sugar uptake and utilization, the mutant cells “sensed” a lack of nutrients, which in turn modified their cellular surfaces to aggregate and survive these sensed starvation conditions.

The transcriptomic assays also revealed, amongst the most up-regulated genes in the mutant, some genes involved in the inter-conversion of homocysteine and methionine. These genes participate in the activated methyl cycle (AMC) a cycle that is involved in AHL biosynthesis. In this cycle, the enzyme S-adenosylmethionine (SAM) synthase synthesized SAM using methionine and ATP as substrates (Fontecave et al., 2004). AHLs are synthesized from precursors derived from fatty-acid biosynthesis and S-adenosylmethionine (SAM) (Val and Cronan, 1998) therefore, it is possible that LuxR402 could also be involved in the regulation of AHLs biosynthesis.

### 3.3. LuxR402, directly or indirectly, regulates the correct assembly of the cell envelopes in *Novosphingobium* sp. HR1a

The down-regulation of the putative regulatory protein involved in lipopolysaccharide (LPS) biosynthesis (HWN72_14815) could orchestrate, at least partially, this cellular surface modification, although more experimental work will have to be carried out to further advance in the regulation cascade. In agreement with the modification in LPS suggested by the transcriptomic results and identified in the physiological experiments, a significantly higher number of peptides related with peptidoglycan biosynthesis and LPS assembly were identified among the extracellular proteins that are more abundant in the wild-type strain than in the mutant strain. In *E. coli*, it has been described that *murG* inactivation (*HWN72_12615*) provokes the inactivation of peptidoglycan biosynthesis and alterations in cell shape (Mengin-Lecreulx et al., 1991), phenotypes that correlate with the lack of detection of peptides that mapped with MurG in the *luxR402* mutant. As in the transcriptomic assays, the carbohydrate-selective porin OprB (HWN72_20595) was also identified at higher numbers amongst the extracytosolic proteins in the wild-type strain than in the mutant strain. The presence of higher amounts of proteins involved in the energy metabolism and cell division in the wild-type than in the mutant strain also supports a higher metabolic rate for *Novosphingobium* sp. HR1a.

In our micrographs of the *luxR402* knockout mutant, we could observe some structures that resembled outer membrane vesicles (OMVs) (Figure 3). OMVs have been involved in environmental mechanisms of adaptation. *Novosphingobium* sp. PP1Y is a bacterial strain that lacks LPS on the outer membrane surface and that forms flocks during the late exponential growth phase (Notomista et al., 2011). The formation of these OMVs has been correlated with the secretion of proteases and glycosylases, possibly involved in acquiring nutrients from extracellular environment (de Lise et al., 2019). In the *luxR402* knockout mutant (whose phenotype resembles that observed for *Novosphingobium* sp. PP1Y) we have observed an increase number of proteins related with polymer degradation (alginate) and also a glycosylase, suggesting that this increase could be a consequence of the “false detection” of nutrient scarcity in the cultures of the mutant strain.

When we challenged the cultures with an antibiotic that targeted cell wall biosynthesis (ampicillin) or with saline stresses, we did not observe significant differences in growth between the mutant and the wild-type strains suggesting that cell envelope integrity was not compromised in the mutant strain. However, addition of 0.5 mM H_2_O_2_ completely inhibits the growth of the mutant, while only retarding the growth of the wild-type, suggesting that cell envelope was less resistant to oxidative stress. This inability can be related with the decreased amount of catalases observed in the *luxR402* knockout mutant (Table 1) and it can be partially supported by the fact that the transcriptomic analyses revealed that four catalases genes (HWN72_02305, HWN72_28310, HWN72_28660 and HWN72_30395) are down-regulated in the mutant strain, although with log_2_FC around 0.6–0.7 (Supplementary Table S1). However, we have demonstrated that the mutant cells have less specific TTC-reducing activity than the wild-type, an ability that is related with the correct conformation of the electron transfer chain, supporting the idea of an abnormal configuration of the cell envelope as the mechanism for H_2_O_2_ susceptibility. Furthermore, the presence of H_2_O_2_ (at 0.5 mM or 0.2 mM) did not modified the expression of the *luxR402* promoter (not shown), supporting that the effect of this stress is an indirect consequence of the alteration in cell envelope configuration.

### 3.4. The presence of some toxic compounds inhibits the *luxR402* gene expression and provokes flocculation

The presence of adverse environmental conditions, in addition to starvation, can also cause auto-aggregation as a defense mechanism against stress (Farrell and Quilty, 2002; Klebensberger et al., 2006; Maganha de Almeida and Quilty, 2016); i.e. *Pseudomonas putida* CP1, although capable of degrading mono-chlorophenols, formed aggregates in the culture media when any of these phenols were in the culture media at high concentrations (Farrell and Quilty, 2002). In our experiments, we observed the formation of these clumps in the presence of salicylate (5 mM), a compound that is toxic for the cells at higher concentrations, although it is metabolized by *Novosphingobium* sp. HR1a.

In *P. aeruginosa* PAO1, that is able to grow with SDS, it was shown that cellular aggregation occurs in response to SDS (Klebensberger et al., 2006). This phenotype is controlled by the signal transduction system SiaABCD that also controls the Psl polysaccharide, the CdrAB two-parter secretion system and the CupA fimbriae formation. SiaA is putative ser/thr phosphatase and SiaD a di-guanylate cyclase and mutants in *siaA* have decreased content of cyclic-diguanosine monophosphate, a cytosolic second messenger (Colley et al., 2016). Furthermore, the flocculation phenotype modulated by SiaABCD that have been described as energy-dependent process; it only occurred under high energy supply, meaning that it is an active process (Klebensberger et al., 2006). In *Novosphigobium* sp. HR1a, we have observed, not only that the stress produced by salicylate induced flocculation of the culture, but also that the expression from the *luxR402* promoter in *Novosphingobium* sp. HR1a was completely eliminated in the presence of this compound. These results supported the idea that LuxR402 controls the flocculation phenotype as a resistance mechanism. By reducing *luxR402* expression in the presence of these toxic compounds, the cells will form clumps that will allow the survival of the strain in the presence of these toxins. The fact that LuxR402 modulates the PTS systems also correlate the ability of the strains to “sense” their energetic status with the flocculation phenotype.

### 3.5. Flocculation as a biotechnological trait

Flocculation is a type of microbial behavior that can be applied in industrial fermentation and wastewater treatment (Ojima et al., 2015). Many microbial species, mainly bacteria, produce extracellular biopolymers that mediate flocculation (Yokoi et al., 1995; Wu et al., 2010; Mabinya et al., 2012; Bisht and Lal, 2019; Jang et al., 2020). Bioflocculants are, in general, environmental friendly (Salehizadeh and Shojaosadati, 2001; Nontembiso et al., 2011), nonetheless, the high production cost has been a limitation to industrial scale production and application (Wan et al., 2013; Dlangamandla et al., 2016; Mohammed and Dagang, 2019). The purpose of the flocculation in biotechnological processes is to be able to remove biomass once the bioprocess has finished and therefore, the bacteria are able to flocculate by themselves and are biotechnologically relevant. By modulating the level of expression of *luxR402*, we would be able to avoid the use of additional flocculants thus decreasing the costs of the biotechnological processes.

## 4. Materials and methods

### 4.1. Strains, plasmids, primers and media used

Strains, plasmids and primers used in this study are described in Supplementary Table S2.

### 4.2. Growth experiments

The strains were grown overnight in LB medium plus the corresponding antibiotic to be used as inocula for the different experiments. For the analysis of the growth of the mutant and wild-type strain, 200 μL M9 minimal medium plus the corresponding carbon source at a final concentration of 10 mM were dispensed into wells of honeycomb microplates (OY Growth Curves AB Ltd., Raisio, Finland). The wells were inoculated with the overnight culture at an initial optical density (DO_660nm_) of 0.1. The cultures were incubated at 28°C with maximal agitation in a FP-1100-C Bioscreen C MBR analyzer system (OY Growth Curves Ab Ltd., Raisio, Finland). Growth was monitored using a type at 30°C with continuous agitation. Turbidity was measured using a sideband filter at 420–580 nm every 60 min for 42 h. Assays were run in duplicate and were repeated for at least three independent experimental rounds. In experiments in which GFP measurements were taken, growth was analyzed in a Varioskan device as shown below.

Congo red was added in minimal solid medium M9-glucose at a concentration of 25 μg mL^−1^ (Vozza et al., 2016). Swarming plates were prepared as described in Kearns (2010). In both cases a drop of 10 μL of the overnight LB-grown cultures were deposited onto the plate and incubated at 30°C for 4 days. Twitching agar plates were prepared as described by Turnbull and Whitchurch (2014). Afterwards the solid medium was punched out of the bottom of the petri dish plate with a sterile 100 μL pipette tip that was previously submerged in the overnight cultures. After 4 days of incubation at 30°C, the agar media was removed and the plates were rinsed with water. The plates were incubated for 5 min at room temperature with crystal violet (0.4% w/v in water), and washed with water previous to the visualization of the biofilms formed by the different strains (Haley et al., 2014). After that, crystal violet was resuspended in 1 mL of 30% acetic acid (v/v) and absorbance was measured at 595 nm.

TTC reduction experiments were performed as indicated in Defez et al. (2017). Bacterial trains were cultivated in M9 minimal media with glucose plus 0.25 mM H_2_O_2,_ till they reached OD_660nm_ ≈ 1. At that time, number of CFUs was analyzed and 300 μL of these cultures were centrifuged, cells were harvested and resuspended in a phosphate buffer containing 24 mM of TTC. After 10 min of incubation at room temperature, cells were again centrifuged and pellets were resuspended in DMSO to solubilize formed formazan. The production of this compound was measured in a spectrophotometer at λ = 510 nm; results were normalized taking into account the number of CFUs.

### 4.3. Microscopy

M9-glucose liquid cultures of *Novosphingobium* sp. HR1a or *luxR402* mutant strains were taken when the OD_660nm_ was around 0.8. The samples were treated with a mixture 1:1 (v/v) of glutaraldehyde (2.5%) and *p*-formaldehyde (2%) in 0.1 M cacodylate buffer (pH = 7.4) for 24 h at 4°C, and analyzed using a variable pressure scanning electron microscopy (Leo1430vp, 2 Zeiss dsm 950, 4 Supra40vp).

### 4.4. Bacteria adhesion to hydrocarbons (BATH)

Cell surface hydrophobicity was measured using the method described by Rosenberg et al. (1980). Briefly, exponentially grown cultures (OD_660nm_ ≈ 0.8–1) were harvested by centrifugation (5,000 rpm, 10 min, 4°C) and washed twice with 0.8% (w/v) NaCl. The cells were then re-suspended in 4 mL of 0.8% NaCl (final OD_660nm_ ≈ 0.6) and 0.6 mL of *n*-hexane were added. Solution was vortexed for 1 min and left for 10 min at room temperature. Afterwards, the OD_660nm_ of the water layer was measured (A). BATH (%) was calculated as (1−A/0.6) × 100.

### 4.5. Construction of the mutant in *luxR402*

Genomic DNA of *Novosphingobium* sp. HR1a was used as template for the amplification of a 497 bp fragment of the gene *orf402* using oligonucleotides 402-F and 402-R (Supplementary Table S2). This fragment was cloned into pMBL™-T plasmid, resulting in plasmid pMBL402. Plasmid pHP45ΩKm (Prentki and Krisch, 1984) was digested with *Bam*HI and *Sca*I. The Ω-Km cassette exscinded from plasmid pHP45ΩKm was extracted from an agarose gel and ligated into pMBL402, previously cut with *Bam*HI. The resulting plasmid pMBL402Km was transformed into *Novosphingobium* sp. HR1a by electroporation. The transformants that had integrated the plasmid into the host chromosome via homologous recombination were selected on LB plus kanamycin plates and checked by Southern blot hybridization (not shown).

### 4.6. Construction of pR402 reporter plasmid

The 295 intergenic region between *orf402* and orf*403* of *Novosphingobium* sp. HR1a was amplified with primers incorporating restriction sites (an *Eco*RI site in the primer designed to meet the 5’end and a *Pst*I site in the primer designed to meet the 3’end). Upon amplification, the DNA was digested with *Eco*RI and *Pst*I and ligated into the medium-copy number pSEVA637 vector (Silva-Rocha et al., 2013), previously cut with the same enzymes. The resulting plasmid pR402 was sequenced to verify the promoter sequence. This plasmid was electroporated into *Novosphingobium* sp. HR1a and/or the *luxR402* knockout mutant. The transformants were selected in LB plates plus gentamycin; individual colonies were grown in liquid media and the plasmid was extracted and digested with *Eco*RI and *Pst*I to verify the incorporation of the plasmid.

The analysis of *luxR402* expression was carried out by microtiter plate-based assays with the strain *Novosphingobium* sp. HR1a (pR402) or *Novosphingobium* sp. HR1a *luxR402* (pR402) knockout mutant using Greiner 96 black-welled plates. The overnight cultures grown in LB were diluted (after washing three times with distilled water) in M9 minimal medium supplemented with different carbon sources (at a concentration of 5 mM) to reach an initial OD_660nm_ of 0.05. Growth (30°C with continuous shaking at 360 rpm) and fluorescence (excited at 485 nm and read at 535 nm) were monitored in a Varioskan LUX Multimode Microplate Reader (Thermo Fisher Scientific Inc., Singapore). The measurements were normalized with the turbidity of the culture.

### 4.7. Complementation of the strain

The promoter region and the complete *luxR402* (1,084 pb) was amplified using primers in which *Eco*RI and *Bam*HI restriction sites were included (Supplementary Table S2) and, the DNA resulting from the amplification was digested with these restriction enzymes and ligated in the expression vector pBBR1MCS-5 (Kovach et al., 1995), previously digested with *Eco*RI and *Bam*HI. The resulting plasmid p402 was sequenced in order to verify the sequence. Plasmid p402 and pBBR1MCS-5 were independently electroporated into *Novosphingobium* sp. HR1a and in the *luxR402* knockout mutant. The transformants were selected in LB plates plus gentamycin (50 μg mL^−1^). Individual colonies were grown in liquid media and the plasmid was extracted and digested with *Eco*RI and *Bam*HI to verify the incorporation of the plasmid. Positive colonies of each strain *Novosphingobium* sp. HR1a (p0), *Novosphingobium* sp. HR1a (p402), *Novosphingobium* sp. HR1a *luxR402* (p0), *Novosphingobium* sp. HR1a *luxR402* (p402) were used in the complementation assay as indicated in the text.

### 4.8. RNA-seq experiments

Five mL of cultures at exponential phase of growth (OD_660nm_ 0.8) grown in M9 minimal medium plus glucose of *Novosphingobium* sp. HR1a and the *luxR402* mutant were centrifuged (6,000 rpm during 10 min) and the pellets were immediately frozen in liquid N2 and stored a −80°C.

Total RNA extraction and preparation of libraries were carried out as in Molina et al. (2021). Briefly, the RNA was extracted using the Trizol method (TRIzol RNA Isolation Reagents, Thermofisher Scientist), followed by DNAse treatment and purification with the RNeasy Mini Kit (Qiagen). One microgram of RNA per sample was used to prepare the sequencing libraries, which were generated by Novogen (Hong Kong). Sequencing procedures and sequencing analysis was also done by Novogen as previously described (Molina et al., 2021). Two independent cultures of each strain were used for the analysis.

### 4.9. Proteomic analysis (iTRAQ)

20 mL sof overnight M9-glucose cultures of *Novosphingobium* sp. HR1a and the isogenic *luxR402* mutant at early stationary phase of growth (OD_660nm_ 1.8–2.1) were vigorously vortexed for 1 min before pelleting the cells by centrifugation (6,000 rpm, 4°C, 10 min). The supernatants were filtrated using a 22 μm filter (syringe filter 0.2 μm, Whatman) and sent for analysis to the proteomic facility at the CNB-CSIC (Madrid, Spain; http://proteo.cnb.csic.es/proteomica). 12 mL of each sample was concentrated using the Amicon^®^ Ultra-4 filter units (Merck Millipore, cutoff MWCO 3,000) by centrifuging 4 mL of the supernatant at 4,000×*g* at 4°C for 30–40 min and the procedure was repeated 3 times. After concentration, the filter membrane was washed with 100 μL of 5% (v/v) SDS buffer. The final protein concentration was measured by a Bradford method compatible with the presence of detergents (A660 nm), protein quantities varied between 6 and 17 μg. The samples were dried and re-suspended in 100 μL of lysis buffer with 5% SDS supplemented with 100 mM Tris (2-Carboxyethyl) phosphine hydrochloride (TCEP) and 200 mM chloroacetamide (CAA) to reduce and alkylate the proteins. The samples were digested with 1 μg of trypsin. Tryptic peptides were cleaned using C18 stage tips and 800–1,000 ng of these peptides (quantified by QBIT) were analyzed by liquid nano-chromatograpy (Thermo Ultimate 3,000) (C18 reverse phase, 75 μm inner diameter and 25 cm length, flux 250 nLmin^−1^, 60 min), coupled to DDA mass spectrometry (Thermo Orbitrap Exploris OE240).

The obtained MS1 and MS2 specters were used to search, using Peaks 7.5, against, a database formed by 6,069 entries of *Novosphingobium resinovorum* SA1 and a list of common laboratory contaminants. Search parameters were set as follows: (i) peptide mass tolerance ±10 ppm for ion precursors and 0.02 Da for fragments masses; (ii) fix modification, cysteine carbamydomethylation as; and (iii) variable modifications, acetyl at the N-terminal and methionine oxidation [Gln- > pyro-Glu (N-term Q), Glu- > pyro-Glu (N-term E)].

We considered the number of total peptides as an estimate of the relative abundance of the protein. Only the proteins identified with more than 6 peptides were considered relevant.

### 4.10. Statistical analysis

Statistical differences between the different categories inside each experiment were determined using SigmaPlot 11 program, using *t*-test analysis or Mann–Whitney Rank Sum Test.

## Data availability statement

The datasets presented in this study can be found in online repositories. The names of the repository/repositories and accession number(s) can be found at: https://www.ncbi.nlm.nih.gov/geo/, GSE223267.

## Author contributions

AS and LM carried out the experimental design and work, and writing the paper. All authors contributed to the article and approved the submitted version.

## Funding

This work was supported by grant number BIO2017-85994-P financed by MCIN/AEI/10.13039/501100011033/ and “ERDF A way of making Europe” and Grant number PID2020-116766GB-I00 financed by MCIN/AEI/10.13039/501100011033.

## Conflict of interest

The authors declare that the research was conducted in the absence of any commercial or financial relationships that could be construed as a potential conflict of interest.

## Publisher’s note

All claims expressed in this article are solely those of the authors and do not necessarily represent those of their affiliated organizations, or those of the publisher, the editors and the reviewers. Any product that may be evaluated in this article, or claim that may be made by its manufacturer, is not guaranteed or endorsed by the publisher.
