# Supplementary figures and images for: LuxR402 of Novosphingobium sp. HR1a regulates the correct configuration of cell envelopes
Source: Front Microbiol. 2023 Jul 27;14:1205860. doi: 10.3389/fmicb.2023.1205860 (PMC10413115; doi:10.3389/fmicb.2023.1205860)

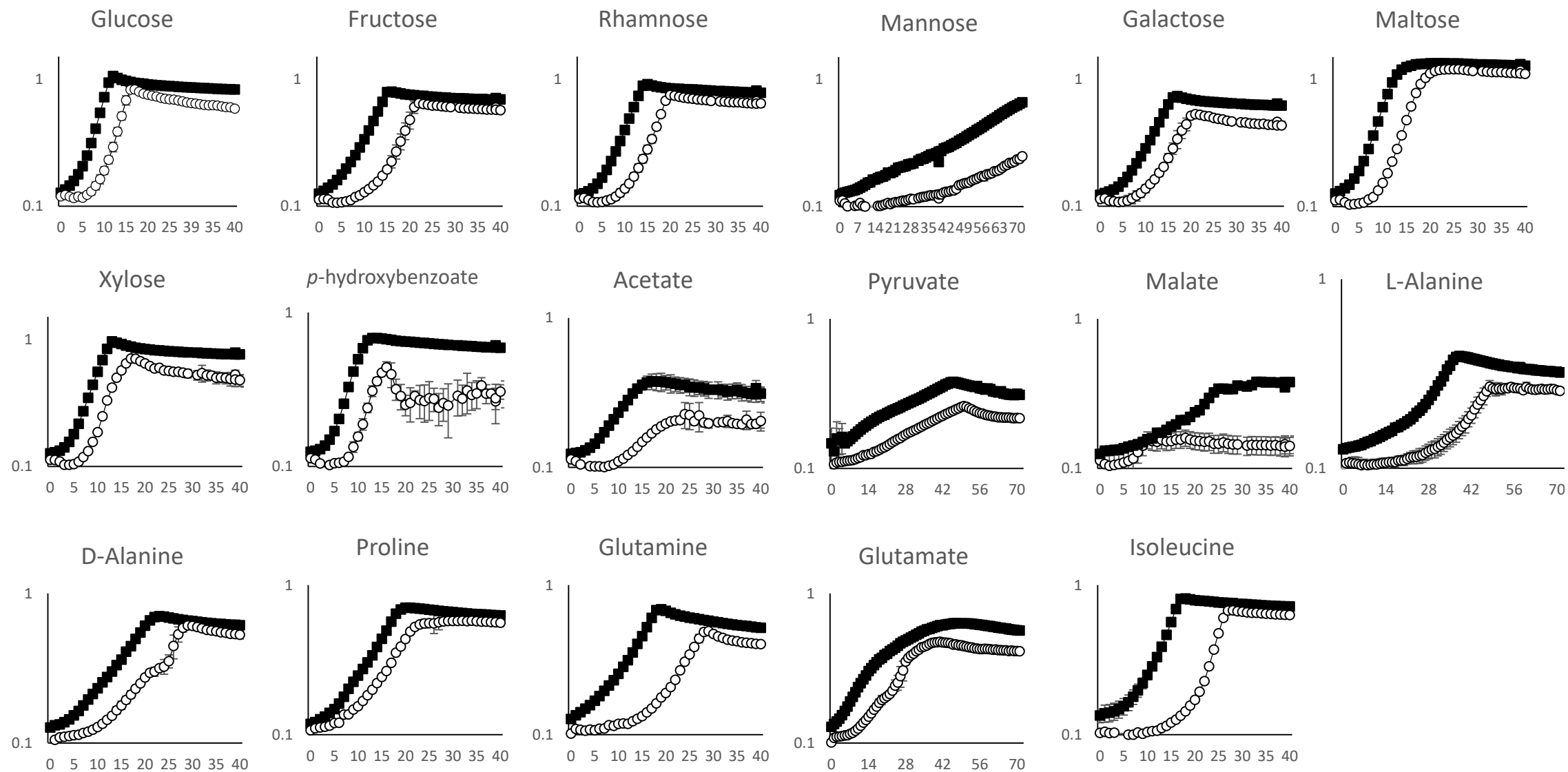

Suppl. Figure 1

Supplement: Supplementary file 3 [file Image_1.pdf]

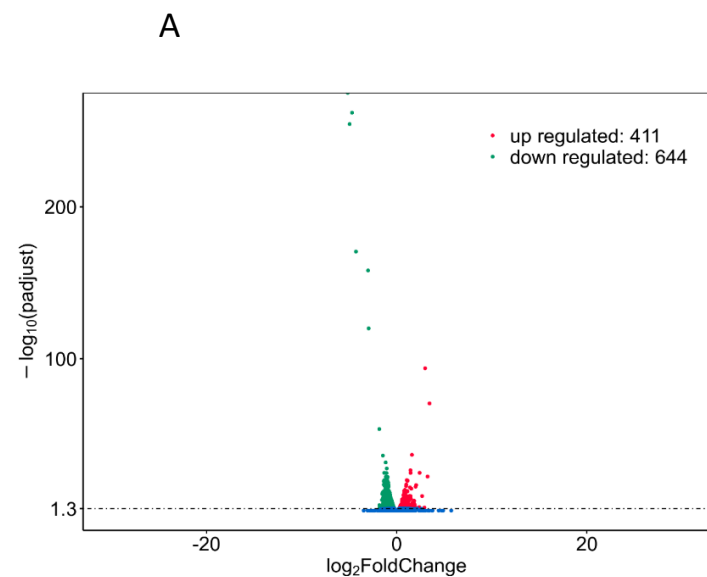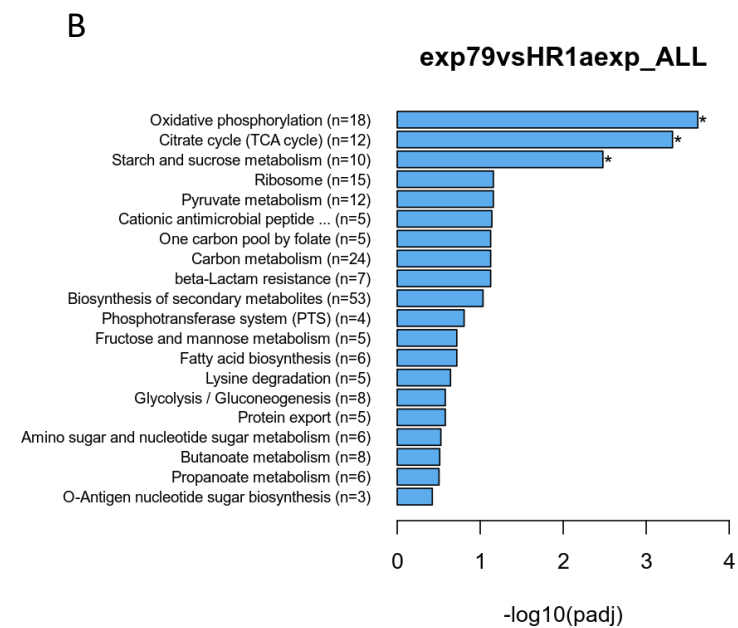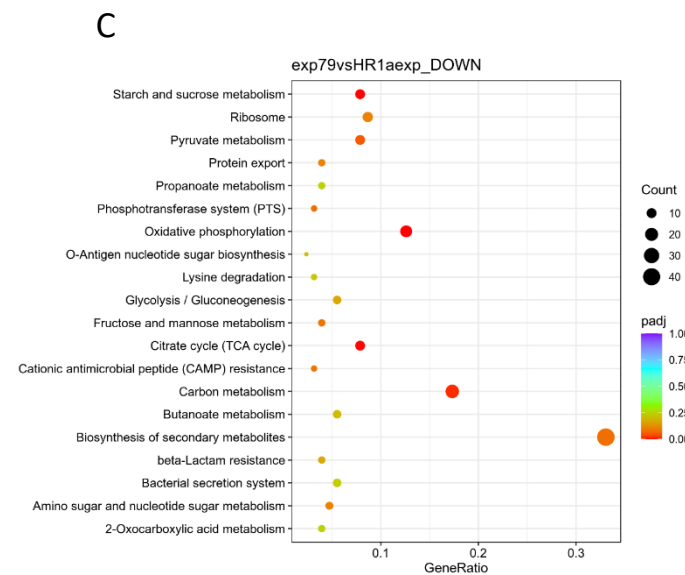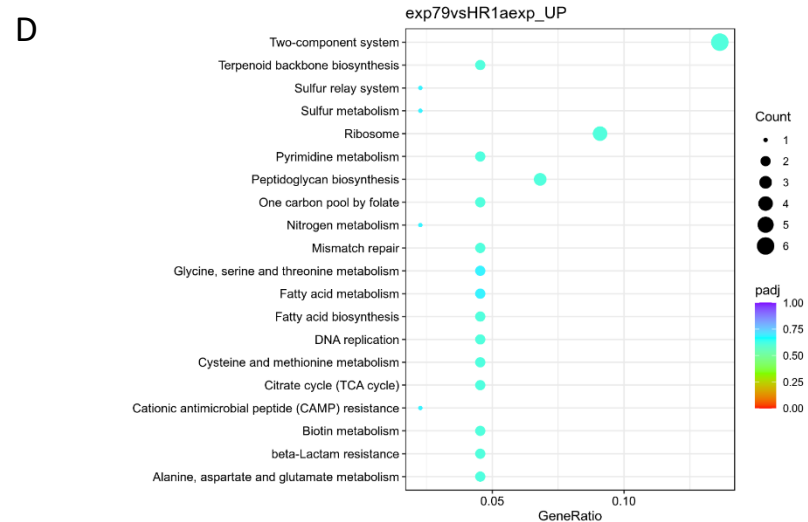

Supplement: Supplementary file 4 [file Image_2.pdf]
